# Integration of Fetal Middle Cerebral Arterial (MCA) Ultrasonography in Acute Fetal-Maternal Hemorrhage

**DOI:** 10.1155/2019/4363054

**Published:** 2019-10-13

**Authors:** Jessian L. Munoz, Maria Schleicher, Natalie Bowersox

**Affiliations:** ^1^Department of OB/GYN, Division of Maternal-Fetal Medicine, University of Texas Health San Antonio, San Antonio, TX, USA; ^2^OB/GYN and Women's Health Institute, Cleveland Clinic, Cleveland, OH, USA

## Abstract

Here we present a case of maternal-fetal hemorrhage characterized by intermittently reassuring fetal testing. Additional testing performed with ultrasound, including middle cerebral artery (MCA) doppler waveforms, confirmed fetal hemorrhage followed by emergent cesarean section. This report highlights the acute usage of MCA dopplers in obstetric decision making. The Newborn required transfusion but otherwise recovered well. MCA may be a useful tool for fetal assessment in Labor and Delivery units.

## 1. Case Report

**Figure 1:**
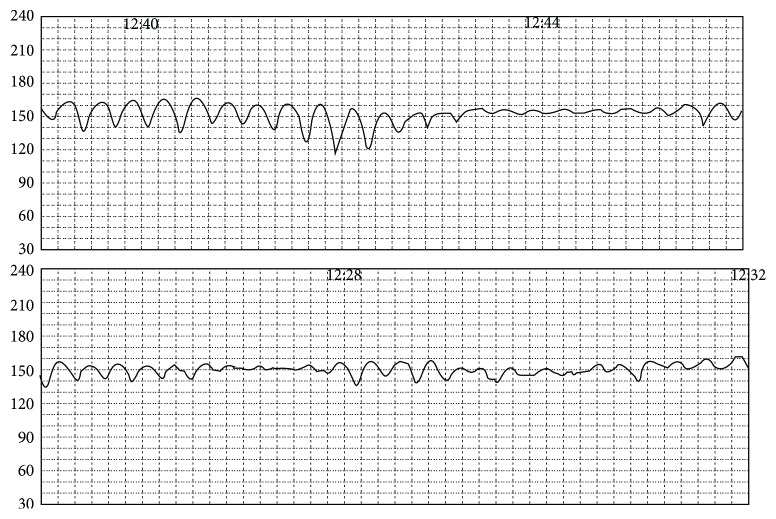
Electronic fetal heart monitoring (EFM). 14 consecutive (7 min upper, 7 min lower) minutes of EFM are presented. Throughout the EFM periods of sinusoidal and category 2 tracings are noted to alternate without persistence of either pattern. Baseline FHR 150 bpm.

**Figure 2:**
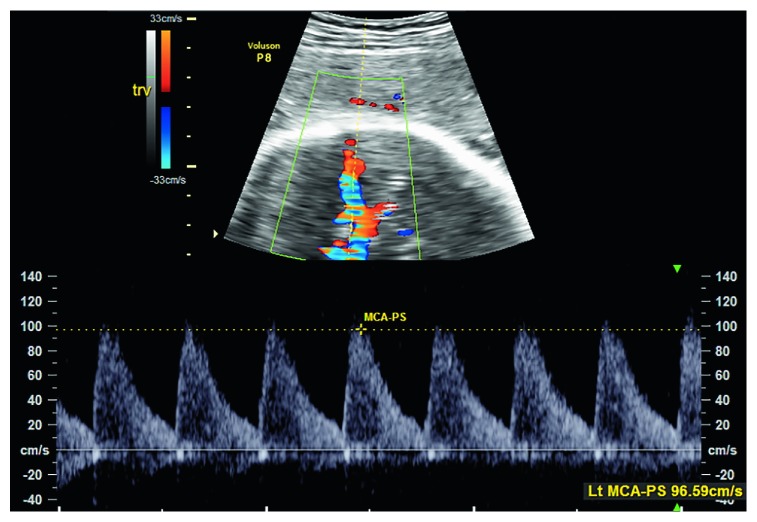
Emergent MCA doppler. Shown is the left MCA doppler obtained by maternal fetal medicine staff at time of fluctuating electronic fetal heart monitoring (EFM). Increased MCA Peak Systolic Velocity (PSV) is indicative of severe fetal anemia.

**Figure 3:**
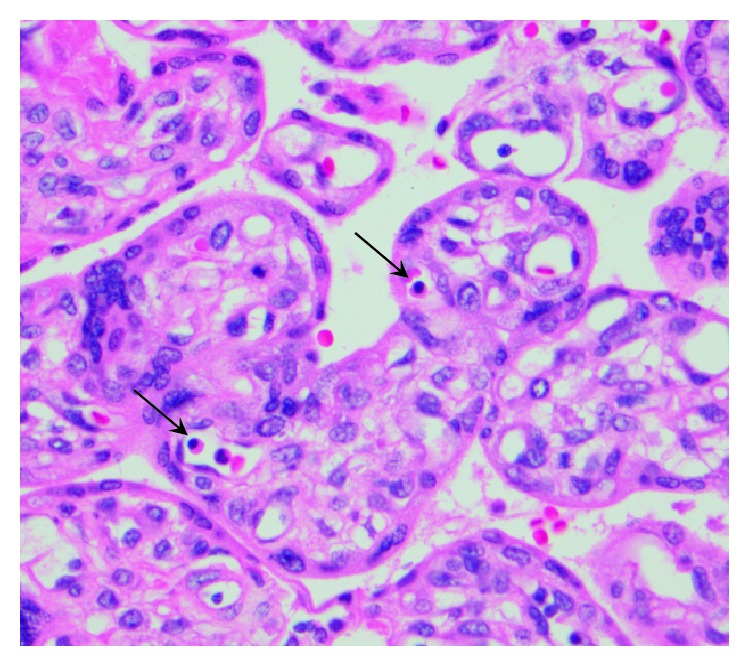
Placental histology. High power (40x) H&E staining of the placental terminal villi. Nucleated red blood cells are noted within the terminal villi (black arrows). The presence of nucleated RBCs shows acute fetal hemorrhage within the placenta.

Patient is a 32-year-old G1P0 at 33 weeks and 1 day gestation that was admitted to the hospital for further evaluation of decreased fetal movement for the two days. Pregnancy was uncomplicated prior to this admission. The patient was started on continuous fetal monitoring along with intravenous fluids. The patient was found to have alternating category II and III tracing (Figure 1). Given the prematurity of the fetus, Maternal-Fetal Medicine was emergently consulted. Bedside ultrasound was performed for amniotic fluid index, biophysical profile, placental appearance and MCA Doppler. Middle cerebral artery peak systolic velocity was 96 cm/s (1.88MoM) (Figure 2). This data was suggestive of fetal anemia most probably secondary to spontaneous feto-maternal hemorrhage. Due to nonreassuring fetal status along with intermittent category-three tracing, the decision was then made to proceed with emergent primary low transverse cesarean section. She then underwent a low transverse cesarean section and delivered a 4 pound, 6 ounce infant female with a one-minute Apgar score of four and a five-minute Apgar score of seven. Umbilical blood gas pH was 6.98. At time of surgery, placenta was noted to be pale in appearance. Placental pathology revealed a 368 g, appropriate for gestational age placenta with increased circulating nucleated red blood cells and focal chronic villitis (Figure 3). Perioperatively, female infant was also noted to be pale appearing; hemoglobin at that time was 2 g/dL. Infant required transfusion of 2 units of packed red blood cells. Kleihauer betke staining revealed 318 mL's of Fetal blood present in maternal circulation. Infant progressed normally throughout admission. Infant is currently 3 years old with no deficits.

## 2. Discussion

Fetal-maternal hemorrhage (FMH) is the presence of fetal blood cells in the maternal circulation prior to the time of delivery. Under normal circumstances, maternal and fetal circulations are kept separate from direct exchange by the placental membrane composed of syncytiotrophoblasts and cytotrophoblasts [1]. Gaseous and micronutrient exchange freely occurs at this interphase. There is a certain degree of minimal blood flow that is considered physiologic and inherent to pregnancy. FMH represents an entity in which supraphysiological amounts of blood extravasate the fetus and enter maternal circulation [2]. The exact amount of fetal blood that constitutes this supraphysiological value is unknown and open to interpretation. Current Rhogam® administration protects against alloimmunization from up to 30 mL of fetal blood. Thus some have used the need for Rhogam administration due to fetal-maternal blood exchange as a definition for FMH [3].

MCA doppler ultrasonography is often used as a screening tool for chronic fetal anemia such as parvo B19 infection or twin anemia-polycythemia syndrome and is not routinely used on labor and delivery units [4]. Inpatient utility may be limited by user skill or comfort but as in the case presented, using MCA dopplers can uncover acute episodes of fetal hemorrhage.

While fetal heart rate monitoring continues to be the initial assessment of fetal wellbeing, advanced supplemental ultrasonography may provide additional tools for clinical management of complex cases or extreme prematurity. In addition, a category III tracing is indicative of fetal acidemia. Ultrasonography offers a non-invasive method for assessing fetal well-being when compared to amniocentesis or cordocentesis [5]. We uncovered 2 reported additional cases of doppler ultrasonography usage for acute hemorrhage [6]. Together these cases advocate for advanced ultrasound training and the incorporation of ultrasonography in the evaluation of acute fetal-maternal hemorrhage.
